# Human testis–specific Y-encoded protein-like protein 5 is a histone H3/H4-specific chaperone that facilitates histone deposition *in vitro*

**DOI:** 10.1016/j.jbc.2022.102200

**Published:** 2022-06-27

**Authors:** Sambit Dalui, Anirban Dasgupta, Swagata Adhikari, Chandrima Das, Siddhartha Roy

**Affiliations:** 1 Structural Biology and Bioinformatics Division, Council of Scientific and Industrial Research (CSIR)-Indian Institute of Chemical Biology, Kolkata, India; 2 Biophysics and Structural Genomics Division, Saha Institute of Nuclear Physics, Kolkata, India; 3 Homi Bhaba National Institute, Mumbai, India

**Keywords:** chromatin, histone H3/H4, histone chaperone, NAP1, TSPYL5, histone deposition, AR, acidic region, BLI, biolayer interferometry, BSA, bovine serum albumin, CAF-1, chromatin assembly factor 1, DSS, 2,2-dimethyl-2-silapentane-5-sulphonate, GST, glutathione-*S*-transferase, HBM, histone-binding–deficient mutant, HBS, Hepes-buffered saline, HEK293T, human embryonic kidney 293T cell line, IP, immunoprecipitation, NAP, nucleosome assembly protein, NAP1L, NAP1-like protein, Ni–NTA, nickel–nitrilotriacetic acid, NLD, NAP-like domain, PDB, Protein Data Bank, SEC–MALS, size-exclusion chromatography coupled with multiangle light scattering, SET, Su(var)3–9, enhancer-of-zeste and trithorax, TAF-1, template-activating factor 1b, TCEP, Tris(2-carboxyethyl)phosphine, TSPY, testis-specific Y-encoded protein, TSPYL5, TSPY-like protein 5, USP, ubiquitin-specific protease

## Abstract

DNA and core histones are hierarchically packaged into a complex organization called chromatin. The nucleosome assembly protein (NAP) family of histone chaperones is involved in the deposition of histone complexes H2A/H2B and H3/H4 onto DNA and prevents nonspecific aggregation of histones. Testis-specific Y-encoded protein (TSPY)–like protein 5 (TSPYL5) is a member of the TSPY-like protein family, which has been previously reported to interact with ubiquitin-specific protease USP7 and regulate cell proliferation and is thus implicated in various cancers, but its interaction with chromatin has not been investigated. In this study, we characterized the chromatin association of TSPYL5 and found that it preferentially binds histone H3/H4 *via* its C-terminal NAP-like domain both *in vitro* and *ex vivo*. We identified the critical residues involved in the TSPYL5–H3/H4 interaction and further quantified the binding affinity of TSPYL5 toward H3/H4 using biolayer interferometry. We then determined the binding stoichiometry of the TSPYL5–H3/H4 complex *in vitro* using a chemical cross-linking assay and size-exclusion chromatography coupled with multiangle laser light scattering. Our results indicate that a TSPYL5 dimer binds to either two histone H3/H4 dimers or a single tetramer. We further demonstrated that TSPYL5 has a specific affinity toward longer DNA fragments and that the same histone-binding residues are also critically involved in its DNA binding. Finally, employing histone deposition and supercoiling assays, we confirmed that TSPYL5 is a histone chaperone responsible for histone H3/H4 deposition and nucleosome assembly. We conclude that TSPYL5 is likely a new member of the NAP histone chaperone family.

Within the eukaryotic nucleus, chromatin exists as a highly compacted dynamic DNA–protein complex, which regulates several critical cellular processes. Chromatin is constituted of functional units called nucleosomes, which in turn are comprised of core histone proteins wrapped around with a 146 base pair DNA in a left-handed superhelix. Each core histone octamer consists of two copies of each H2A, H2B, H3, and H4 (1). DNA-dependent cellular events like replication, transcription, and damage repair machinery require access to the free DNA, which is regulated by the disassembly and reassembly of nucleosomes (2, 3, 4, 5). Histones in their free state are prone to form non-nucleosomal aggregates. Hence, newly synthesized histones need constant governance during their import into the nucleus and subsequent repositioning onto DNA. Histone chaperones, which are a diverse group of proteins, bind to histones at the surfaces required for nucleosome formation (6, 7, 8, 9) and shield them from non-nucleosomal interaction with the DNA (10). They are a multifaceted group of proteins with distinct structural and functional properties, which sometimes exhibit low or no sequence similarity with each other. However, with their involvement in histone folding and import, they are further implicated in histone *de novo* deposition, recycling, and exchange to maintain chromatin plasticity (8, 11). Some of the prominent histone chaperones that have been well studied and characterized include nucleoplasmin (12), chromatin assembly factor 1 (CAF-1), ant silencing factor 1 (Asf1) (13, 14, 15, 16), facilitates chromatin transcription (FACT) (17, 18), Spt2 (19, 20), regulator of Ty1 transposition 106 (Rtt106) (21, 22, 23), and nucleosome assembly protein 1 (NAP1). NAP family of histone chaperones remains conserved from yeast to humans and are involved in cell-cycle regulation, transcription, replication, gene silencing, and apoptosis (24, 25). Structures of the yeast NAP1 (24), along with Vps75 (26, 27, 28) and human SET (Su(var)3–9, enhancer-of-zeste and trithorax)/TAF-1 (template-activating factor 1b) (29), reveal that they have an N-terminal long helix and a globular domain at the C terminus. In a dimeric state, the long helices interact with each other and form the dimerization interface, whereas the two globular domains positioned at each end form the earmuff-like structure. *In vivo* studies have previously shown that yeast NAP1 not only can bind to histone H2A/H2B (24, 25, 30) but also can bind to H3/H4 *in vitro* (31). Most of the previous evidence suggested that the NAP1 dimer binds to a single H3/H4 dimer (31, 32). However, recent reports indicate that two H3/H4 dimers may also bind a single NAP1 dimer (10, 30, 33).

Testis-specific Y-encoded protein (TSPY)–like protein 5 (TSPYL5) belongs to the testis-specific Y-encoded-like (TSPYL) protein family in humans and is found to be variably expressed in different tissues. Previous studies have shown that the TSPYL5 gene remains hypermethylated in nearly all the primary gliomas and is a marker of the suppressor of cell growth. TSPYL5 also undergoes aberrant hypermethylation-mediated silencing in melanoma, and increased methylation is correlated with disease progressions like gastric cancer and hepatocellular carcinoma (34, 35). Furthermore, TSPYL5 was shown to modulate the growth of adenocarcinoma by regulating the cellular levels of p21(WAF1/Cip1), and the PTEN (phosphatase and tensin homolog)/AKT pathway in turn helps to grow resistance to cytotoxic agents such as γ-radiation (36). Recent reports suggest that TSPYL5 prevents ubiquitin-specific protease 7 (USP7)–dependent polyubiquitination of POT1 and its subsequent proteasomal degradation in ALT+ cells (37). TSPYL5 has also been reported to interact with USP7 to reduce the tumor suppressor activity of p53 and prevent oncogene-induced senescence (38, 39). TSPYL5-mediated activation of endoplasmic reticulum stress–induced apoptosis suppresses cell proliferation, migration, and invasion of tumor cells in colorectal cancer (40). Although tumor suppressor activity of TSPYL5 has also been reported in several cancers (41), no significant information is available regarding the role of TSPYL5 in chromatin-mediated processes. In this study, we identified human TSPYL5 as a novel member of the NAP histone chaperone family. Our data indicate that TSPYL5 preferentially binds to histones H3/H4 both *in vitro* and *ex vivo*. We have employed biochemical and biophysical approaches to study the basis of TSPYL5 binding to histone H3/H4. In addition, we characterized TSPYL5–H3/H4 complex and their binding stoichiometry through size-exclusion chromatography coupled with multiangle light scattering (SEC–MALS) and chemical cross-linking assays. Furthermore, we established the role of TSPYL5 as an H3/H4-specific histone chaperone using histone deposition and plasmid supercoiling assays.

## Results

### TSPYL5 exhibits homology with the NAP histone chaperone family

**Figure 1:**
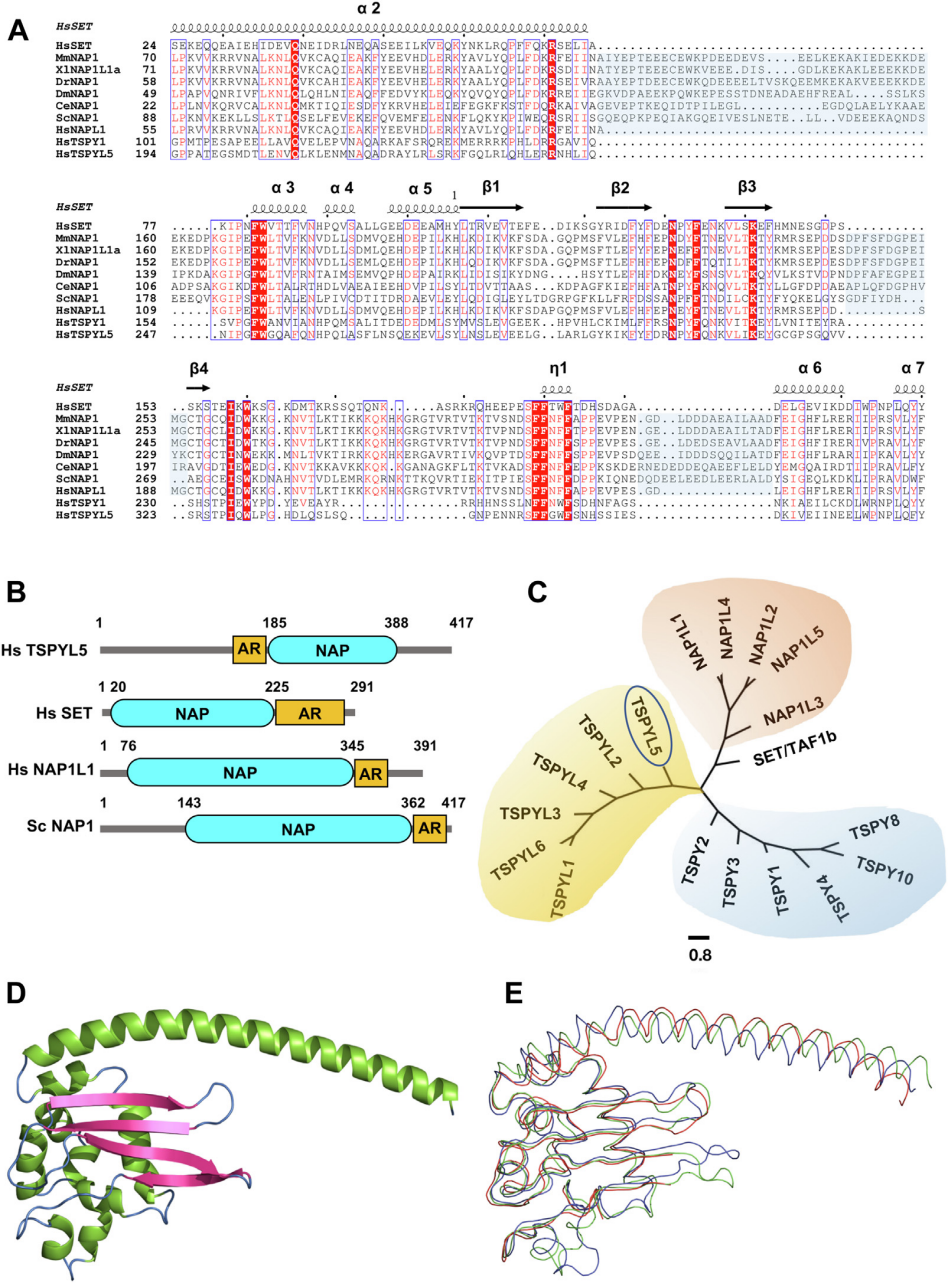
**TSPYL5 shares significant sequence homology with NAP family proteins.***A*, multiple sequence alignment of human TSPYL5 (Q86VY4) with members of NAP protein family across different species. The different NAP family members (UniProt accession ids within parenthesis) included in the analysis were ScNAP1 (P25293), CeNAP1 (Q19007), DmNAP1 (Q9W1G7), DrNAP1 (Q803X7), XlNAP1 (Q4U0Y4), MmNAP1 (P28656), HsNAP1L1 (P55209), HsTSPY1 (Q01534), and HsSET/TAF-1B (Q01105). Identical residues are labeled in *red*. NAP1 accessory region is highlighted in *blue*. Secondary structure prediction of SET/TAF-1B (PDB ID: 2E50) is shown above the multiple sequence alignment where alpha helix and beta strand are indicated. *B*, domain architecture of human TSPYL5, SET/TAF-1B, NAP1L1, and yeast NAP1. NAP domains are highlighted in *cyan*, and acidic regions (ARs) are highlighted in *yellow*. *C*, phylogenetic tree depicting different NAP-like proteins in human. MEGA-X and Fig-Tree Software packages were used for the analysis. The scale bar is indicated in the figure. *D*, molecular model of TSPYL5 (198–398) in cartoon representation using AlphaFold. Secondary structure is represented in color codes—helix (*green*), beta sheet (*pink*), and loop (*blue*). *E*, superimposition of the C-alpha trace of earmuff domains of TSPYL5 (*green*), RoseTTAFold (*blue*), and SET/TAF-1B (*red*). NAP, nucleosome assembly protein; NAP1L1, NAP1L NAP1-like protein; SET, Su(var)3–9, enhancer-of-zeste and trithorax; TAF-1B, template-activating factor 1b; TSPYL5, testis-specific Y-encoded protein–like protein 5.

NAPs, which are characterized by the conserved α/β-earmuff motif across different species, facilitate *in vitro* assembly of nucleosomes (24). To find out possible proteins with sequence and structural similarity with TSPYL5, we employed multiple sequence alignment using NAP family proteins across different species. The alignment result suggests that TSPYL5 is a homolog of NAP family proteins that remains conserved among eukaryotes (Fig. 1*A*). The C-terminal stretch of TSPYL5 (198–398) shows significant sequence similarity with the SET/TAF-1B and other NAP family proteins harboring the signature NAP fold, indicating the presence of NAP-like domain (NLD) in TSPYL5. However, TSPYL5-NLD significantly lacks the extended NAP1 accessory region present in most of the NAP family proteins across different species. The majority of the NAP family proteins share a common acidic region (AR) comprising mostly of aspartate and glutamate residues in their C-terminal stretch. Unlike other NAP family proteins, the AR of TSPYL5 is present at the N terminus of the NLD (Fig. 1*B*). Interestingly, compared with other eukaryotes, the NAP proteins in the human genome exist as multiple diverse subfamilies. To understand the similarity between the different NAP family proteins in humans, we generated a phylogenetic tree using a maximum likelihood approach. Tree data show the divergence of human NAP proteins into three distinguished subfamilies—NAP1-like proteins (NAP1L), TSPY proteins, and TSPY-like proteins, whereas human SET/TAF-1b diverged and remained distinct from the aforementioned subfamilies (Fig. 1*C*). To obtain a detailed understanding of the structure of TSPYL5-NLD, we took recourse to molecular modeling using the AlphaFold software package (DeepMind). From the AlphaFold prediction, TSPYL5 was found to form a “headphone” structure consisting of two distinct regions. Region-I contained an extended helix (α-1) spanning 50 amino acid residues, which form the “head” segment of the headphone structure. Region-II consists of the globular α/β “earmuff” segment of the headphone. This globular domain contains four antiparallel β-strands (β1–β4) and with four short α-helices (2, 3, 4, 5). β-strands form a β-sheet, and the short α-helices are arranged on the back side of the β-sheet headphone (Fig. 1*D*). We also generated the molecular model of TSPYL5 using the RoseTTAFold server (https://robetta.bakerlab.org/), which is similar to the AlphaFold model (RMSD of the C_α_ atom is 2 Å). To check whether TSPYL5 showed structural similarity with the previously reported crystal structure of human SET/TAF-1B (Protein Data Bank [PDB] ID: 2E50), we superimposed them, which revealed that TSPYL5 shared a similar structural fold with SET/TAF-1B (RMSD is 2.2 Å) (Fig. 1*E*).

### TSPYL5 interacts specifically with histone H3/H4 dimer

**Figure 2:**
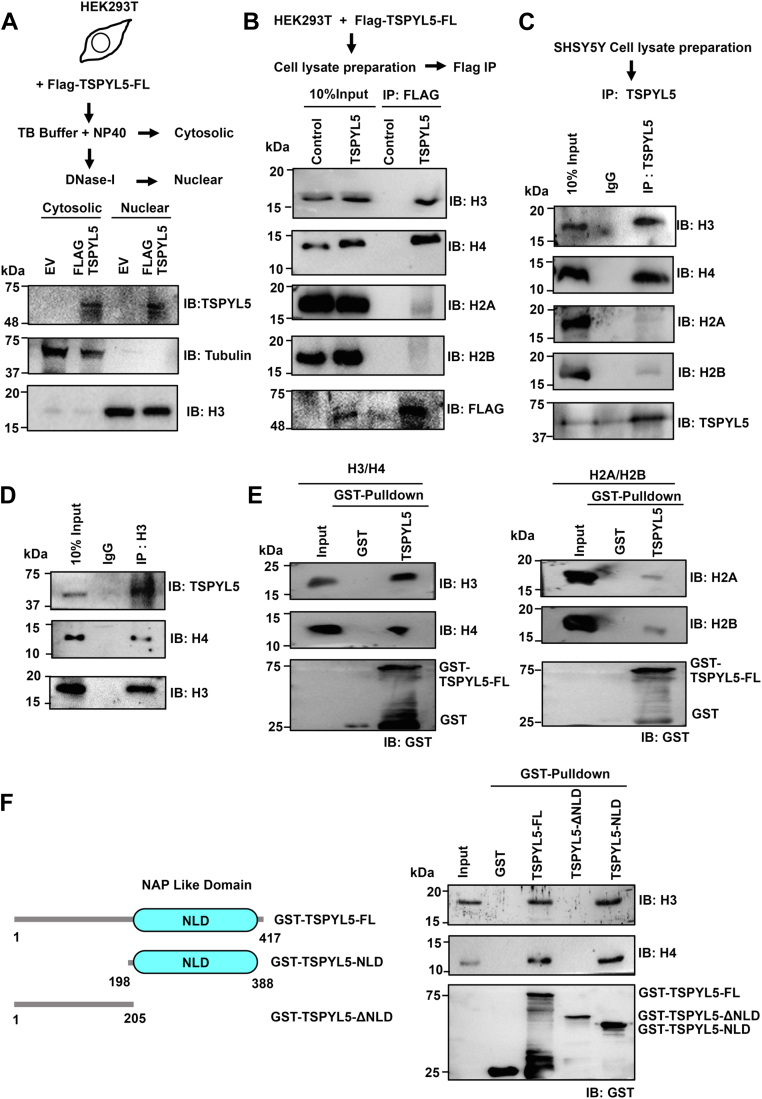
**TSPYL5 interacts with histone H3/H4.***A* and *B*, HEK293T cells were transfected with empty vector (EV; as a negative control) and FLAG-TSPYL5 and subsequently separated into cytosolic and nuclear fractions. The localization of TSPYL5 was detected using Western blotting. *A*, tubulin and H3 were used as cytosolic and nuclear markers. *B*, immunoprecipitation of FLAG-TSPYL5 using anti-FLAG antibody was followed by immunoblotting with all four core histone αH2A, αH2B, αH3, and αH4 antibodies. *C* and *D*, endogenous association of TSPYL5 with core histones in SH-SY5Y cells. *C*, TSPYL5 was immunoprecipitated from SH-SY5Y cells using anti-TSPYL5 antibody and subsequently immunoblotted with αH2A, αH2B, αH3, and αH4 antibodies. *D*, reciprocal immunoprecipitation with αH3 antibody was performed followed by immunoblotting with αTSPYL5 antibody. *E* and *F*, *in vitro* interaction of GST-TSPYL5-FL or its different domains with histone H3/H4 or H2A/H2B dimer. *E*, GST pull-down assay was performed followed by immunoblotting with αH2A, αH2B, αH3, and αH4 antibodies. *F*, subsequently, GST-conjugated TSPYL5-FL, TSPYL5 NAP-like domain (NLD) and ΔNLD proteins were pulled down and probed with αH3 and αH4 antibodies. Domain architecture of different constructs used in the pull-down assay has been shown in the *left panel*. GST, glutathione-*S*-transferase; HEK293T, human embryonic kidney 293T cell line; NAP, nucleosome assembly protein; TSPYL5, TSPY-like protein 5.

NAP family member proteins have been reported to be involved in multiple pathways, including nucleocytoplasmic shuttling, chromatin assembly, remodeling, replication, transcription, gene silencing, and apoptosis (24, 25, 42, 43, 44, 45, 46), which requires cytoplasmic or nuclear localization or both. To look into the localization of TSPYL5, we performed a cell fractionation assay using FLAG-TSPYL5–transfected human embryonic kidney 293T (HEK293T) cell line. TSPYL5 was found to be present in both cytosolic and nuclear fractions (Fig. 2*A*). Nuclear localization of TSPYL5 however raises the possibility of it having a role in a chromatin context, which has not yet been studied. Previously, NAP family histone chaperones have been widely reported to interact with both histones H2A/H2B and H3/H4 (25, 31). To identify histone-interacting partners of TSPYL5, we performed a FLAG immuno-pulldown assay using overexpressed FLAG-TSPYL5 in HEK293T cells followed by immunoblotting with different histone antibodies. Results showed that TSPYL5 specifically interacts with histones H3 and H4 but not with histones H2A and H2B (Fig. 2*B*). Next, we wanted to look into the histone interaction of endogenous TSPYL5. Since endogenous expression of TSPYL5 is significantly higher in the SH-SY5Y cell line compared with HEK293T, we performed an immuno-pulldown assay involving endogenous TSPYL5 in SH-SY5Y cells. Pulled samples using anti-TSPYL5 antibodies were immunoblotted with different histone antibodies, whereby TSPYL5 was found to interact specifically with H3 and H4 (Fig. 2*C*). We further confirmed the interaction of TSPYL5 with histone H3/H4 by performing a reverse immunoprecipitation (IP) with an anti-H3 antibody (Fig. 2*D*). To check if TSPYL5 directly interacts with histones H3/H4, we further performed a glutathione-*S*-transferase (GST) pull-down assay using recombinant TSPYL5 and histones. *In vitro* pull-down assay indicates that TSPYL5 directly interacts with histone H3/H4 but not with histone H2A/H2B (Fig. 2*E*). Next, to identify the region of TSPYL5 involved in H3–H4 interaction, we expressed and purified different deletion constructs of TSPYL5 and checked for their H3–H4 binding ability through a GST pull-down assay. TSPYL5-NLD binds with histone H3/H4 with an intensity comparable to that of TSPYL5-FL, whereas TSPYL5-ΔNLD fails to interact (Fig. 2*F*). Thus, TSPYL5-NLD is essential for its interaction with histone H3/H4.

### Mapping of TSPYL5 and histone H3/H4–interacting surface

**Figure 3:**
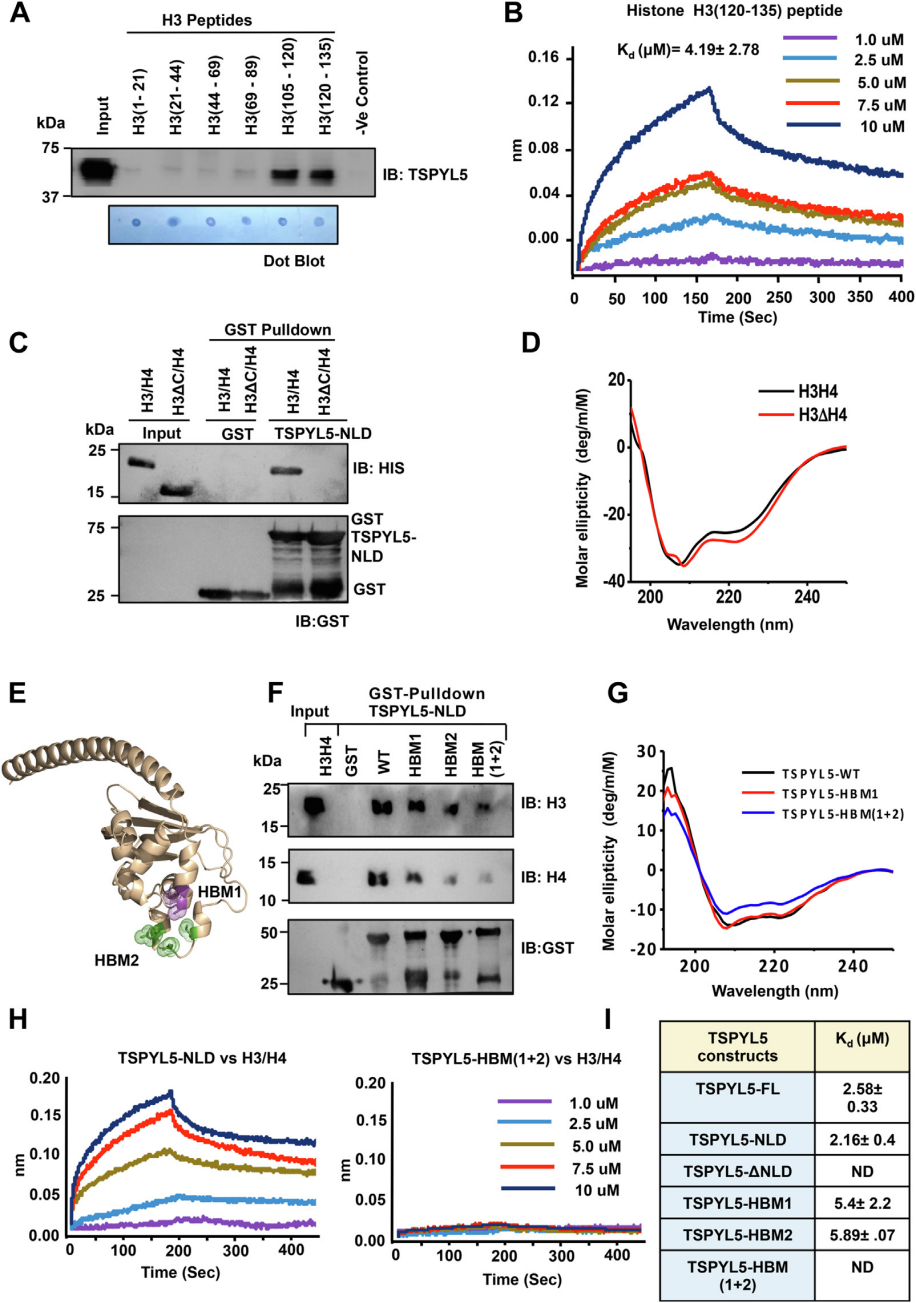
**Fine mapping of TSPYL5–H3/H4 interaction.***A*, peptide pull-down assay of the recombinant TSPYL5-NLD with biotinylated histone H3 peptides. Streptavidin beads were used for histone peptide pull-down assay, and Western blotting with αTSPYL5 antibody was performed. Peptide dot blot was performed to show equivalent amount of peptides were used in the assay. *B*, binding affinity of TSPYL5-NLD with histone H3 C-terminal peptide (120–136 amino acids) using biolayer interferometry (BLI). The analyte concentrations of TSPYL5 are shown in different colors. *C*, *in vitro* GST pull-down assay of the TSPYL5-NLD with recombinant His-tagged histone H3/H4 or H3ΔC-term/H4 was performed and subsequently blotted with αHis and αGST antibodies. *D*, far-UV CD spectra of histone His-H3/H4 or His-H3ΔC-term/H4 constructs. *E*, histone-binding–defective mutants (HBM1 in *pink* and HBM2 in *green*) are highlighted in *stick representation* in the TSPYL5 *cartoon diagram*. *F*, *in vitro* interaction of GST-TSPYL5-NLD, HBM1, HBM2, or HBM (1 + 2) with histone H3/H4. GST pull-down assay was performed followed by immunoblotting with αH3 and αH4 antibodies. *G*, far-UV CD spectra of WT and HBM constructs of TSPYL5-NLD. *H* and *I*, binding affinity of TSPYL5-NLD or HBM (1 + 2) with histone H3/H4 using BLI. *H*, the analyte concentrations of TSPYL5-NLD or HBM (1 + 2) are shown in different colors. *I*, *K*_*d*_ values for the interaction between histone H3/H4 and TSPYL5-FL, TSPYL5-NLD, or its different mutants are represented in the table. GST, glutathione-*S*-transferase; NLD, NAP-like domain; TSPYL5, TSPY-like protein 5.

We next attempted to fine-map the regions involved in histone H3/H4–TSPYL5 interaction. To identify the TSPYL5-binding surface of histone H3, we generated different biotinylated H3 peptide fragments encompassing full-length histone H3. We performed an *in vitro* peptide-pulldown assay to score the interaction between H3 peptides and TSPYL5-NLD and observed that histone peptides involving the C-terminal H3 tail, encompassing both 105 to 120 and 120 to 136 regions, showed significant interaction with TSPYL5-NLD (Fig. 3*A*). To quantify the affinity of TSPYL5-NLD toward histone H3 C-terminal region, we employed biolayer interferometry (BLI) and confirmed that TSPYL5-NLD binds to histone H3 C-terminal tail (120–136) with a significantly higher affinity (dissociation constant; *K*_*d*_ = 4.19 ± 2.78 μM) compared with other histone H3 peptides (Fig. 3*B*). To further investigate if the C-terminal tail of H3 was sufficient for TSPYL5 interaction, we generated and purified histone H3 C-terminal tail deleted protein (H3ΔC), which lacks the last 21 residues. We then employed a GST pull-down assay using histone H3-WT and H3ΔC. The assay indicates a significant loss of interaction of histone H3ΔC with TSPYL5 compared with H3-WT (Fig. 3*C*). To check whether H3 C-terminal tail deletion had any effect on its conformation, we performed CD spectroscopy with histone H3-WT and H3ΔC-terminal mutants and did not observe any significant structural change upon the deletion (Fig. 3*D*). To map the residues of TSPYL5 that are involved in histone H3/H4 binding, we constructed three different histone-binding–deficient mutants (HBM), namely HBM1 (S268A/E270A), HBM2 (S340A/N343A/E345A), and HBM (1 + 2) (S268A/E270AS340A/N343A/E345A). These mutations have been highlighted in the TSPYL5-NLD model (*cartoon representation*) (Fig. 3*E*). Next, we performed GST pull-down assay, where HBM (1 + 2) exhibited a significant reduction in H3/H4 binding concerning WT (Fig. 3*F*). Subsequently, in order to confirm that the structural conformation of TSPYL5 remains unaltered upon mutations, we performed CD spectroscopy and found no significant conformational changes between WT and HBM mutants (Fig. 3*G*). We further quantified the binding affinity of TSPYL5 toward histone H3/H4 using BLI. TSPYL5-FL and NLD showed significant affinity toward histone H3/H4 with *K*_*d*_ = 2.58 ± 0.33 and 2.16 ± 0.40 μM, respectively. However, HBM (1 + 2) showed no detectable binding to H3/H4. The affinity of TSPYL5 and other mutants toward histone H3/H4 was also investigated using BLI kinetics, and *K*_*d*_ values were summarized (Fig. 3, *H* and *I*).

### Determination of molecular stoichiometry of TSPYL5–H3/H4 complex

**Figure 4:**
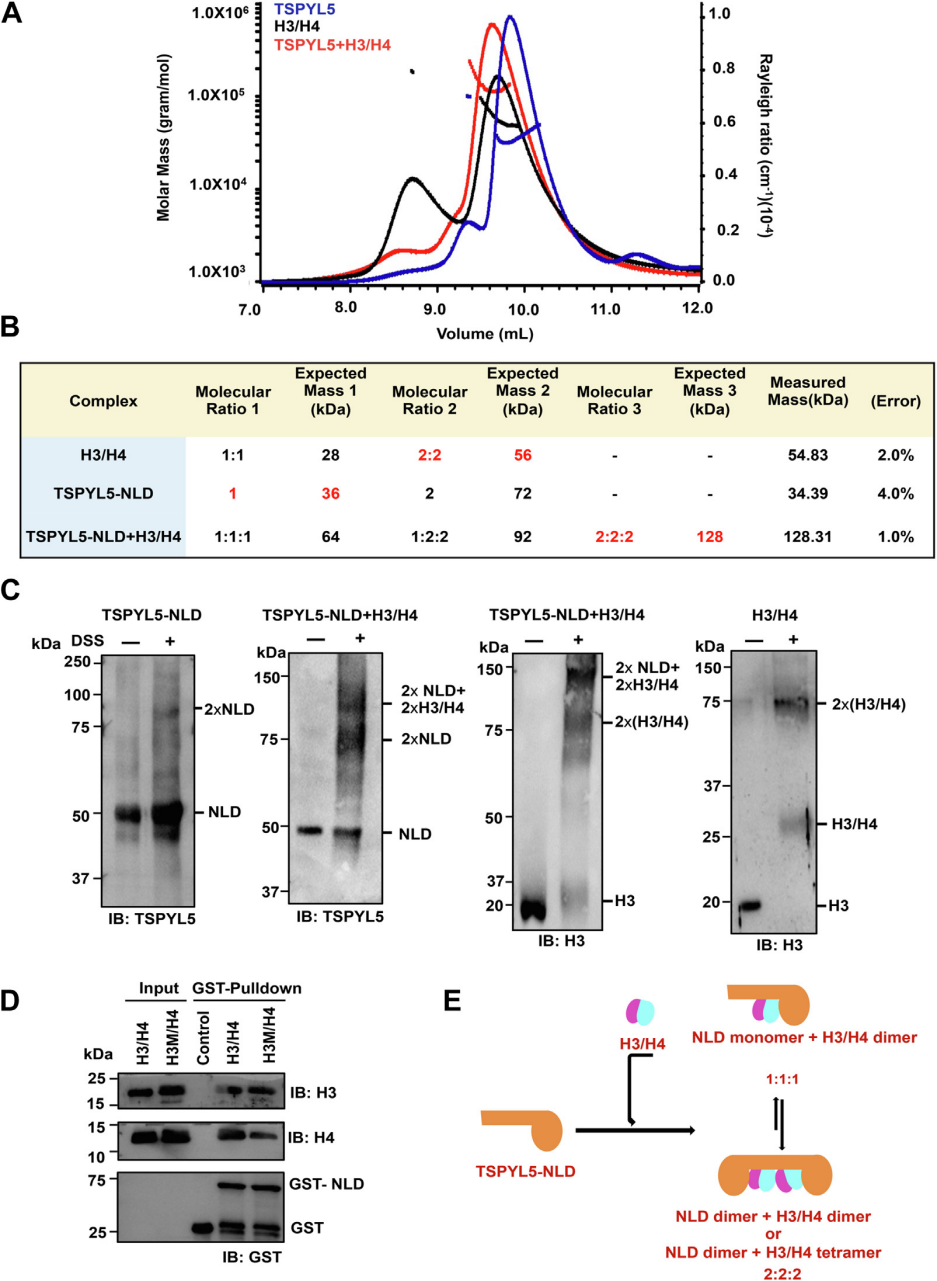
**Molecular characterization of TSPYL5–H3/H4 complex.***A*, SEC–MALS analysis showing normalized refractive index (RI) trace of histone H3/H4 (*black*), TSPYL5-NLD (*blue*), and TSPYL5-NLD–H3/H4 complex (*red*). *B*, molar mass measurements by SEC–MALS (marked in *red*) and the theoretical molecular weight predictions (marked in *black*) of each complex are represented in the table. *C*, DSS crosslinking performed with TSPYL5-NLD or H3/H4 alone or after incubation with TSPYL5-NLD and H3/H4 followed by Western blot analysis. In each case, lane 1 shows control without DSS; lane 2 shows sample incubated with 2.5 mM DSS. Species appearing upon crosslinking are marked. *D*, *in vitro* GST pull-down assay of the TSPYL5-NLD with recombinant histone H3/H4 or H3M/H4 was performed and subsequently blotted with αH3, αH4, and αGST antibodies. Tetramer-disruptive histone H3 construct (H3M) harbors C110E, L126R, and I130E mutations. *E*, schematic representation of binding modes for TSPYL5–H3/H4 complex. DSS, 2,2-dimethyl-2-silapentane-5-sulphonate; GST, glutathione-*S*-transferase; NLD, NAP-like domain; SEC–MALS, size-exclusion chromatography coupled with multiangle light scattering; TSPYL5, TSPY-like protein 5.

To gain further insights into the molecular stoichiometry of the TSPYL5–H3/H4 cocomplex in solution, we used SEC–MALS to measure the molecular mass of the individual proteins as well as the complex. The measured mass of TSPYL5 was obtained at 34.39 (4% error) and 73.92 kDa (2.6% error), respectively. The corresponding expected monomer and dimer mass of TSPYL5 was 36 and 72 kDa. This indicated that TSPYL5 in high salt conditions exists predominantly as a monomer to a lesser extent as a dimer. In a similar high salt condition, H3/H4 molecular mass was measured at 54.83 kDa (2% error, expected histone H3/H4 tetramer mass was 56 kDa). Interestingly, in the case of the TSPYL5–H3/H4 complex, we obtained a molecular mass of 128.31 kDa, suggesting the molar stoichiometry of the complex to be TSPYL5:H3:H4 (2:2:2) (Fig. 4, *A* and *B*). We further performed 2,2-dimethyl-2-silapentane-5-sulphonate (DSS)–mediated protein crosslinking experiments to confirm the presence of the TSPYL5–H3/H4 complex in solution. Upon crosslinking, TSPYL5 and H3H4 together yielded a higher molecular weight species between 150 and 100 kDa, which was significantly absent when TSPYL5-NLD and H3/H4 were crosslinked individually. Western blot results using anti-H3 and anti-TSPYL5 antibodies confirmed the presence of the TSPYL5–H3/H4 complex (Fig. 4*C*). SEC–MALS and solution crosslinking studies indicate that the TSPYL5 dimer forms a complex with two H3/H4 dimers or one tetramer. To find out if TSPYL5 differentiates between H3/H4 dimer and tetramer, we generated a tetramerization-disruptive mutant (H3M/H4) with C110E/L126R/I130E mutation and performed a GST pull-down assay. TSPYL5 was found to bind equally with H3/H4 dimer (involving H3M/H4) and H3/H4 tetramer indicating that TSPYL5 does not differentiate between histone H3/H4 dimer and tetramer (Fig. 4*D*). Collectively, these results indicate that one TSPYL5 dimer can bind with two histone H3/H4 dimers or a single H3/H4 tetramer (Fig. 4*E*).

### TSPYL5 binds to DNA *in vitro*

**Figure 5:**
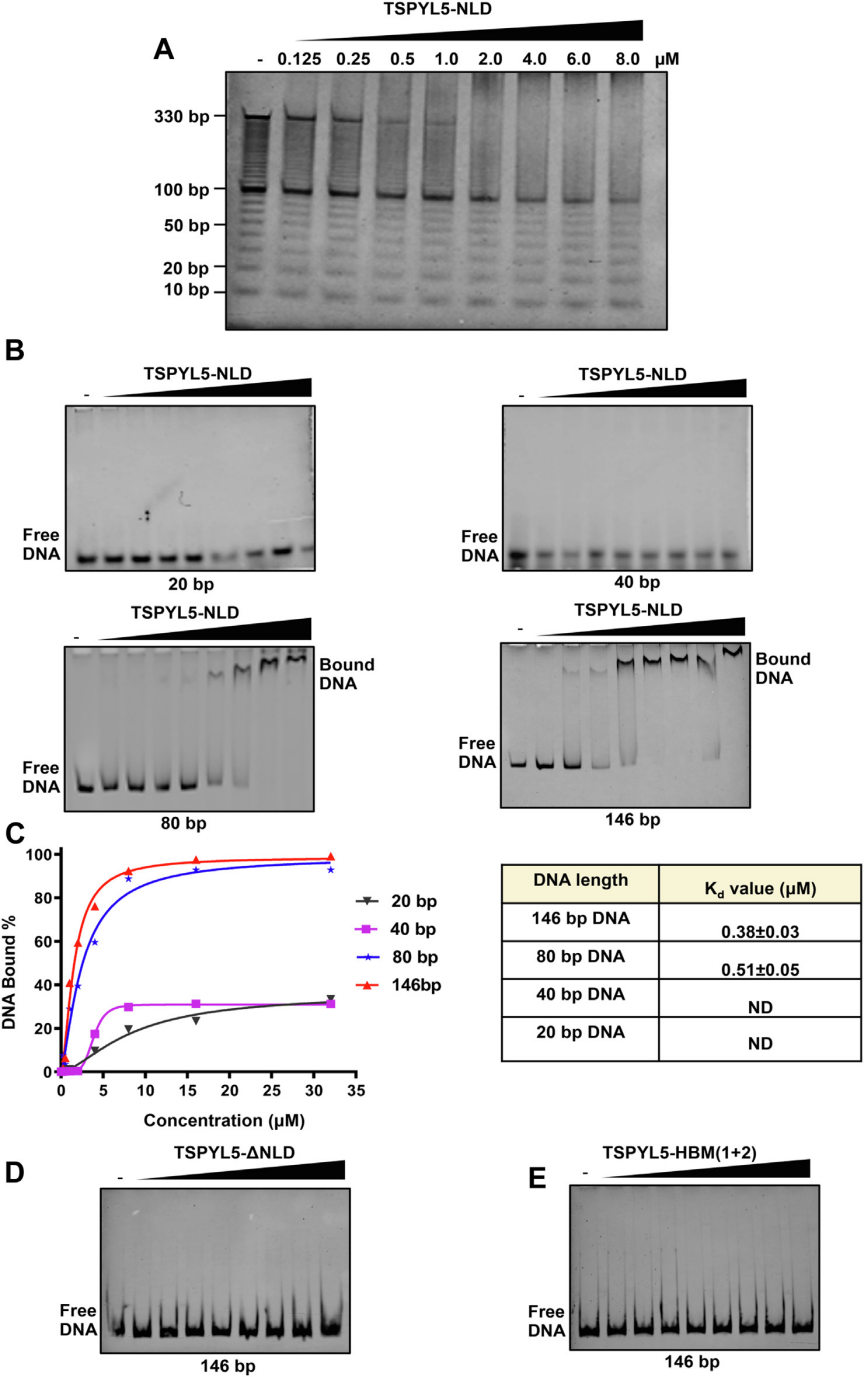
**TSPYL5 binds to DNA.***A*, EMSA showing TSPYL5-NLD binding to free DNA fragments of varying lengths (10–330 bp). *B*, EMSA showing binding of TSPYL5-NLD using 20, 40, 80, and 146 bp DNA, respectively. Increasing amounts of TSPYL5 (0.06, 0.125, 0.25, 0.5, 1, 2, 4, 8, 16, or 32 μM) were mixed with 1 μM DNA. *C*, corresponding binding curves of TSPYL5-NLD to 20, 40, 80, and 146 bp DNA interaction. The binding intensity is quantified by DNA depletion upon interaction with TSPYL5-NLD. *D* and *E*, EMSA showing binding of 146 bp DNA to increasing concentration of TSPYL5-ΔNLD (*D*) and HBM (1 + 2) (*E*). Free DNA and TSPYL5-bound DNA are indicated in all the aforementioned experiments. HBM, histone-binding–deficient mutant; NLD, NAP-like domain; TSPYL5, TSPY-like protein 5.

Histone chaperones, apart from their ability to interact with histones, also have been reported to bind DNA to regulate DNA-mediated processes (47, 48). To check if TSPYL5 has DNA-binding ability, we used an EMSA using different DNA fragments ranging from 10 to 330 base pair (bp) as a substrate, where TSPYL5-NLD was found to bind to DNA above 80 bp length but had no binding with the shorter fragments of DNA (Fig. 5*A*). To further investigate if the specific length of DNA fragment was a prerequisite for TSPYL5 binding, we performed EMSA with 20, 40, 80, and 146 bp dsDNA substrates individually and analyzed the binding affinity of TSPYL5 toward them. We found that TSPYL5 shows no significant binding toward 20 and 40 bp DNA. On the other hand, it showed a high affinity toward 80 and 146 bp DNA (Fig. 5*B*). Interestingly, while a minimum of 80 bp DNA is required to dock H3/H4 tetramer, 146 bp DNA is required to assemble a complete nucleosome. Thus, TSPYL5 binding to these longer DNA fragments indicates that it may have a role in DNA-mediated histone deposition process. To further quantify the DNA-binding affinity of TSPYL5, we measured the substrate DNA depletion in EMSAs, and the binding curve was plotted against increasing concentration of TSPYL5. From the binding data, TSPYL5 shows strong binding toward 80 and 146 bp of DNA with *K*_*d*_ values of 0.5 and 0.38 μM, respectively (Fig. 5*C*). To find out if NLD is sufficient for the DNA binding of TSPYL5, we used TSPYL5-ΔNLD and found no detectable DNA binding. This indicates that NLD is essential and sufficient for the DNA-binding activity of TSPYL5 (Fig. 5*D*). To explore if the histone-binding residues of TSPYL5 were also involved in its DNA binding, we performed EMSA using histone-binding–deficient mutant TSPYL5-HBM (1 + 2). We observed no significant DNA binding, which suggests that the DNA-binding surface of TSPYL5 may overlap with its histone-binding surface (Fig. 5*E*).

### TSPYL5–H3/H4 complex facilitates histone deposition and plasmid supercoiling *in vitro*

**Figure 6:**
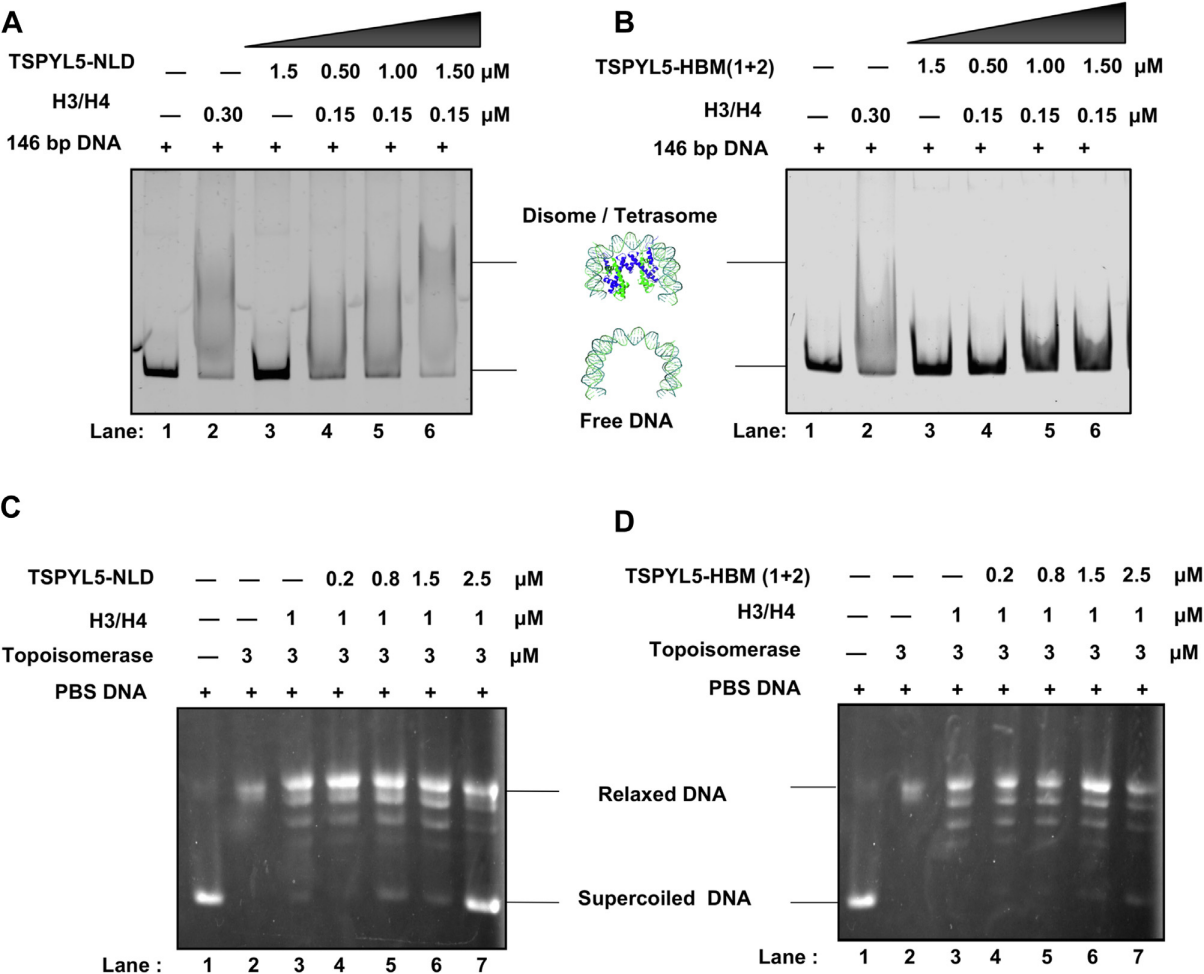
**TSPYL5 promotes *in vitro* histone deposition and plasmid supercoiling.***A* and *B*, histone deposition assay performed with 146 bp linear DNA (Widom 601 sequence), H3/H4, and TSPYL5-NLD or TSPYL5-HBM (1 + 2). Samples run in 7% native-PAGE and stained with GelGreen. Lanes 1 and 2 show free DNA input and tetrasome input, respectively; lane 3 shows TSPYL5-NLD (*A*) or TSPYL5-HBM (1 + 2) (*B*) with free DNA. Lanes 4 to 6 show free DNA incubated with H3/H4 with increasing concentrations of TSPYL5-NLD (*A*) or TSPYL5-HBM (1 + 2) (*B*) as indicated in the figure. *C* and *D*, plasmid supercoiling assay performed with plasmid DNA and topoisomerase I, histone H3/H4, and TSPYL5-NLD or TSPYL5-HBM (1 + 2). Samples were resolved with native agarose gel electrophoresis. Lane 1 shows supercoiled plasmid DNA input; lane 2 shows DNA relaxed by topoisomerase I; lane 3 shows relaxed DNA incubated with histones only; and lanes 4 to 7 show prerelaxed DNA with topoisomerase I, incubated with histone H3/H4, and increasing concentrations of TSPYL5-NLD (*C*) or TSPYL5-HBM (1 + 2) (*D*) as indicated in the figure. HBM, histone-binding–deficient mutant; NLD, NAP-like domain; TSPYL5, TSPY-like protein 5.

To determine if TSPYL5 acts as a *bona fide* histone H3/H4 chaperone, we performed an *in vitro* histone deposition assay. Upon incubation, TSPYL5-NLD in increasing concentration was able to deposit H3/H4 on to linear DNA substrate (Fig. 6*A*). On the other hand, histone-binding–deficient mutant TSPYL5-HBM (1 + 2), having impaired association toward histone H3/H4 *in vitro*, was unable to deposit histones (Fig. 6*B*). To investigate if TSPYL5 could facilitate DNA supercoiling upon histone deposition, we performed a plasmid supercoiling assay using topoisomerase I-treated relaxed plasmid DNA. Increasing concentrations of TSPYL5-NLD resulted in efficient supercoiling of plasmid DNA, suggesting that TSPYL5 may have a role in promoting DNA supercoiling upon histone deposition. However, the TSPYL5-HBM (1 + 2) failed to facilitate plasmid supercoiling *in vitro* (Fig. 6*C*). Together, these results suggest that histone-binding residues of TSPYL5 are critical for its histone deposition and plasmid supercoiling activity.

## Discussion

Chromatin assembly involves a two-step process in which a histone H3/H4 dimer is deposited first onto the DNA followed by two H2A/H2B dimers, assisted by different histone chaperones (49, 50). NAP family of histone chaperones are conserved from yeast to higher eukaryotes. In humans, NAP family proteins have diverged into different subfamilies—including NAP1-like (NAP1L1–5) (51), testis-specific protein Y-encoded (TSPY1–10) (39), TSPY-like (1–6), and SET/TAF-1B (29). Despite sharing little sequence similarity, members of the NAP family (24) exhibit high structural conservation among each other. Yeast NAP1, the first reported member of the NAP protein family, has been majorly described as an H2A/H2B-specific histone chaperone (10, 24, 25, 31, 33, 46, 52). Crystal structure of yNAP1 bound to H2A–H2B (53) together with *in vitro* and *in vivo* functional studies provides molecular evidence of NAP1 binding to H2A/H2B. In contrast, we found that TSPYL5 specifically interacts with histone H3/H4 and not with H2A/H2B in both overexpressed and endogenous conditions. Using recombinant proteins, we further confirmed the direct interaction of TSPYL5 with histone H3/H4. Interestingly, Vps75, which is a yeast homolog of NAP1, and human SET/TAF-1B also show binding preference toward histone H3/H4 over H2A/H2B (7, 27, 28). To identify the histone-interacting region of TSPYL5, we next generated a construct encompassing the C-terminal NLD of TSPYL5 and another construct with the TSPYL5 N-terminal stretch including its AR. NLD was found sufficient for H3/H4 binding, thus indicating that TSPYL5 interacts with H3/H4 *via* its NLD and not through its N-terminal region.

Although multiple NAP family members in humans have been reported to interact with histones, their binding surfaces have not been well studied. Our results revealed that TSPYL5 utilizes the loops, connecting the small helices located at the globular C-terminal NLD, for its histone binding. The involved loops were found to be present on the surface of the earmuff domain. On the other hand, it was intriguing to explore the specific region of histone H3 involved in TSPYL5 binding. We found that the H3 C-terminal tail exhibited strong interaction with TSPYL5. This was further validated using an H3 C-terminal tail deletion, which abolished the TSPYL5 interaction. In contrast, yNAP1 has been found to interact with the N-terminal tail of histone H3 *in vitro* (31). This indicates that the binding modes of histones to different NAP family proteins may involve different binding regions.

We determined the binding stoichiometric ratio of the TSPYL5–H3/H4 complex to be 2:2:2 based on the obtained molecular mass of the complex in solution, which suggests that a TSPYL5 dimer can bind with either two H3/H4 dimers or a single H3/H4 tetramer. Using an H3/H4 tetramer–disruptive mutant, we found that TSPYL5 did not differentiate between H3/H4 dimer and tetramer binding. The formation of a stable TSPYL5–H3/H4 complex of 128 kDa was further confirmed by cross-linking assay. Histone H3/H4 binding preference as exhibited by TSPYL5 is similar to Vps75, which interacts with both dimeric and tetrameric forms of histone H3/H4. Thus, it seems likely that TSPYL5 engages with histones H3/H4 in a similar way as Vps75 (7, 28), which assembles two H3/H4 dimers during nucleosome assembly or is involved in capturing the H3/H4 tetramer evicted during chromatin disassembly in replication-independent and replication-dependent processes.

Histone chaperones, which are directly involved in the DNA-mediated histone deposition process, display DNA-binding properties alongside their histone-interacting ability. Yeast CAF-1, which is involved in nucleosome assembly during replication, shows DNA-binding properties through the coiled-coil and winged helix domain regions of the Cac1 subunit (47, 48). Moreover, Rtt106 and HIRA, which are involved in the direct deposition of different histone H3 variants, bind DNA directly (54, 55, 56). Our findings denote that TSPYL5 has similar DNA-binding property with a specific affinity toward 80 bp and higher length DNA fragments but not with its shorter counterparts. It is noteworthy that yCAF-1 also shows a similar mode of DNA binding. Moreover, our biochemical assays demonstrate that TSPYL5 promotes histone H3/H4 deposition and plasmid supercoiling *in vitro*, thereby confirming its histone chaperone activity. This suggests that TSPYL5 may have a direct role in DNA-mediated nucleosome assembly process. In addition, TSPYL5 has been previously shown to interact with USP7 (37, 38). Interestingly, USP7 has been shown to negatively regulate UHRF1-mediated histone H3 ubiquitination (57, 58). In this context, it may be worthwhile to study the role of TSPYL5 in modulating USP7-mediated histone deubiquitination by its histone H3/H4 chaperone activity. In summary, our results established TSPYL5 as a new member of the human NAP histone chaperone family and confirm it as a histone H3/H4–specific chaperone.

## Experimental procedures

### Cloning and site-directed mutagenesis

TSPYL5 full length, NLD (198–388), and ΔNLD (1–203) were cloned in the pGEX-6p-1 vector (GE Healthcare; catalog no.: GE28-9546-48) with N-terminal GST tag for bacterial expression. TSPYL5-NLD was also cloned in the pETite N-His-SUMO expression vector (Lucigen; catalog no.: 49003-2) using the Expresso T7 cloning system and modified by adding a PreScission protease recognition site for removal of the tags. All the point mutations were generated using QuickChange Site-directed mutagenesis kit (Stratagene; catalog no.: 200524). Histones H3 and H4 were PCR amplified from complementary DNA isolated from HEK293T cells and cloned sequentially in both the multiple cloning site of the pETDuet-1 vector. Histone H3 has an N-terminal His tag, whereas H4 was untagged. Histone H3/H4 tetramer–disruptive mutant (H3M) was also generated in the H3–H4 pETduet1 vector using site-directed mutagenesis according to previously described methods (59, 60). H3 C-terminal tail–deleted construct (H3ΔC-term, 1–114) was also prepared in pETite N-His vector using the T7-His cloning system (Lucigen). All the clones were confirmed by sequencing.

### *In vitro* protein expression and purification

Chemically competent Rosetta(DE3) pLysS cells (Novagen; catalog no.: 70956) were transformed with different TSPYL5 constructs. Cells were grown in a 37 °C shaker incubator till an absorbance at 600 nm reached 0.8 and then induced using 1 mM IPTG (Gold BioI-2481C100) at 18 °C for 16 h. GST-tagged proteins were purified in the GST affinity column and subsequently eluted using reduced glutathione. For biophysical assays, GST proteins were then cleaved by PreScission protease. His-SUMO-tagged TSPYL5-NLD was expressed similarly as mentioned previously. Protein was trapped with nickel–nitrilotriacetic acid (Ni–NTA) bead (Bio-Rad; catalog no.: 780-0800), eluted, and purified on Superdex 200 Increase 16/600 GL gel filtration column in Hepes-buffered saline (HBS) buffer (20 mM Hepes [pH 7.5], 500 mM NaCl, 1 mM Tris(2-carboxyethyl)phosphine [TCEP], 5% glycerol, and 1 mM PMSF). Histone H3/H-WT and H3M/H4 pETDuet-1 constructs were expressed in BL21(DE3) pLysS cells (Thermo Fisher Scientific; catalog no.: C606010) and induced with IPTG at 37 °C for 4 h. Recombinant His-tagged human histones H3, H3ΔC-term, and H4 were expressed in BL21(DE3) pLysS cells followed by purification and refolding following standard procedures (61). Eluted His-tagged H3/H4 was further purified on gel filtration column using high salt HBS buffer (20 mM Hepes [pH 7.5], 2 M NaCl, 1 mM TCEP, 5% glycerol, and 1 mM PMSF). All purified proteins were concentrated, flash-frozen, and stored at −80 ^o^C until further use.

### Antibody generation

Polyclonal anti-TSPYL5 antibody was raised using purified recombinant TSPYL5-NLD protein. An amount of 200 mg of purified recombinant TSPYL5-NLD was injected subcutaneously into New Zealand white rabbit using Freund complete adjuvant in a 1: 1 ratio. This was followed by five booster doses at every 2-week intervals after the first injection. The booster doses were given using Freund’s incomplete adjuvant. Serum was obtained from blood collected from the sixth bleed and finally, purified TSPYL5 antibody was obtained by affinity chromatography. The specificity of the generated anti-TSPYL5 antibody was checked by assessing their binding to various concentrations of recombinant TSPYL5 protein by Western blot.

### Cell culture and cell fractionation

HEK293T and SHSY5Y cells were grown and maintained in Dulbecco’s modified Eagle’s medium (Gibco) at a 37 °C incubator supplied with 5% CO_2_. Dulbecco’s modified Eagle’s medium was supplemented with 10% fetal bovine serum (Gibco), 1% antibiotic–antimycotic (Gibco), and 1% essential amino acids (Gibco). Untransfected and FLAG-TSPYL5–transfected HEK293T cell pellets were resuspended in hypotonic buffer (10 mM Hepes [pH 7.4], 10 mM NaCl, 6 mM MgCl_2_, 2 mM DTT, 0.05% NP-40, 2 mM PMSF, and 1× protease inhibitor cocktail), incubated on ice for 1 h, and centrifuged at 3000 rpm for 5 min. The supernatant fraction was collected as a cytosolic fraction. The pellet fraction was treated with DNase I in nuclear extraction buffer (20 mM Hepes [pH 7.4], 420 mM NaCl, 1.5 mM MgCl_2_, 0.2 mM EDTA, 1 mM DTT, 20% glycerol, and 0.5% NP-40) and incubated at 37 °C for 30 min followed by centrifugation at 13,000 rpm for 10 min at 4 °C. The supernatant fraction thereafter collected was marked as a nuclear fraction, and the pellet was discarded. Fractionation was analyzed by Western blotting using anti-TSPYL5, antitubulin (Bio-Rad; catalog no.: MCA78G) as a cytosolic marker, and anti–histone H3 (Abcam; catalog no.: ab10799) as a nuclear marker.

### Western blot analysis

For preparing whole cell lysates, cells were resuspended in Laemmli buffer (4% SDS, 20% glycerol, and 120 mM Tris–HCl [pH 6.8]) (62) and sonicated before analyzing by Western blot. Samples were run on 11% or 15% SDS-PAGE and transferred on polyvinylidene difluoride membrane followed by blocking with 5% bovine serum albumin (BSA) in 1× Tris-buffered saline with 0.1% Tween-20 and probed with specific antibodies.

### IP

Cells were crosslinked with 4% paraformaldehyde in PBS for 15 min at room temperature, lysed with IP buffer (50 mM Hepes [pH 7.5], 150 mM NaCl, 1.5 mM MgCl_2_, 1 mM EDTA, 1% Triton X-100, 5% glycerol, 1 mM DTT, and 2 mM protease inhibitor cocktail), and incubated on ice for 1 h followed by sonication. Lysates were then centrifuged, collected supernatant was precleared with immunoglobulin G antibody, and IP was performed with FLAG (Sigma; catalog no.: F1804), H3 (Abcam; catalog no.: ab10799), and TSPYL5 antibodies. After washing thoroughly with the same buffer, IP samples were analyzed by Western blotting.

### *In vitro* protein–protein interaction studies

GST pulldown was performed where GST-fusion proteins along with histones were incubated in an equal molar ratio in GST-IP buffer (50 mM Hepes [pH 7.5], 150 mM NaCl, 0.05% NP-40, 1 mM DTT, 5% glycerol, and 1 mM PMSF). Preblocked Glutathione Sepharose (GST) beads (GE Healthcare; catalog no.: 17-0756-01) were used for pull down, and beads were washed in the same buffer and analyzed by Western blotting.

### BLI study for kinetic assay

BLI was used to study protein–protein binding kinetics (63) using the Octet-RED system (Forté Bio). For various assays, recombinant His-tagged histones H3/H4 or H2A/H2B were immobilized on Ni–NTA Biosensors (Forté Bio NTA; catalog no.: 18-5102) in binding buffer (50 mM Hepes [pH 7.5], 150 mM NaCl, 0.05% NP-40, 1 mM DTT, 5% glycerol, and 0.5 mg/ml BSA) and TSPYL5 WT and histone-binding–deficient mutants were used as analyte with increasing concentration. All the data were recorded in triplicates. Reference subtraction, Savitsky–Golay filtering, and local fitting of kinetic rate association and dissociation of the collected data were analyzed using Forté Bio’s software (version 11x).

### Peptide pull-down assay

Biotinylated histone H3 peptides were purchased from GL-Biochem: H3 (1–21), H3 (21–44), H3 (44–69), H3 (69–89), H3 (105–120), and H3 (120–135). Biotinylated histone H3 peptides were incubated with GST (control) and GST-TSPYL5 in peptide IP buffer (50 mM Tris–HCl [pH 7.5], 200 mM NaCl, 0.05% NP-40, and 1 mM DTT) at 4 °C overnight. The next day preblocked streptavidin beads (GE Healthcare; catalog no.: 17-5113-01) were added to the samples to bind for 2 h at 4 °C. Bead-bound samples were washed in IP buffer and analyzed by Western blot.

### TSPYL5–H3/H4 complex purification

His-tagged H3/H4 in pETDuet-1 and His-SUMO-tagged TSPYL5-NLD were coexpressed in Rosetta(DE3) pLysS cells and purified by His-tagged affinity chromatography using Ni–NTA beads. Proteins were eluted using 500 mM imidazole in high salt HBS buffer. Eluted samples were loaded on Enrich SEC70 column using Next-Gen FPLC system (Bio-Rad) in high salt HBS buffer (20 mM Hepes [pH 7.5], 2 M NaCl, 1 mM TCEP, and 5% glycerol) for purification. Fractions containing the complex were pooled and concentrated up to 10 mg/ml using Amicon centrifuge filter units (Millipore; catalog no.: ACS510012) and maintained at 4 °C or flash-frozen and stored at −80 °C for further use.

### DSS cross-linking experiments

DSS cross-linking experiments were performed using previously described methods (60). Briefly, TSPYL5-NLD, H3/H4, and TSPYL5-NLD–H3/H4 complex were incubated with a 1.5 mM DSS crosslinker (Thermo Fisher Scientific; catalog no.: 21655) for 30 min at 25 °C in cross-linking buffer (20 mM Hepes [pH 7.5], 150 mM or 300 mM NaCl, and 1 mM TCEP). Reactions were quenched by the addition of 50 mM Tris–HCl (pH 8.0) for 15 min at 25 °C, and samples were analyzed in 12% SDS-PAGE, transferred to polyvinylidene difluoride membranes, and incubated against H3, H4, and TSPYL5 antibodies. To perform this experiment, we incubated His-SUMO–tagged TSPYL5-NLD and H3/H4 alone or with DSS and resolved crosslinked complexes by SDS-PAGE followed by Western blot.

### SEC–MALS of protein complexes

Purified TSPYL5-NLD–H3/H4 complex was analyzed using SEC–MALS connected with Enrich SEC 70 column equilibrated in high salt HBS buffer. The light scattering intensity and differential refractive index of the column eluate were recorded using a DAWN HELEOS8+laser photometer and Optilab T-rEX differential refractometer (Wyatt Technology), respectively. The weight average molecular mass of the sample peaks in the eluate was determined as a measure of the combined data from both detectors using ASTRA software, version 7.3.2, using 0.186 ml/g value of dn/dc (specific refractive index increment).

### EMSAs

TSPYL5-NLD, HBM (1 + 2), and ΔNLD were used in increasing concentration (0.06, 0.125, 0.25, 0.5, 1, 2, 4, 8, 16, or 32 μM) and incubated with 20, 40, 80 bp, and 146 bp dsDNA or 10 bp ladder (Invitrogen; catalog no.: 10821-015), respectively, in EMSA buffer (10 mM Hepes [pH 7.5], 200 mM NaCl, 1 mM TCEP, 5% glycerol, and 0.5 mM EDTA). The samples were maintained on ice for 30 min and then shifted at 25 °C for 20 min before analysis. The binding reactions were analyzed on a 6% native 1× Tris–glycine (pH 8.3) PAGE using 1× Tris–glycine running buffer. The gel was stained with GelGreen (GoldBio; catalog no.: G745). Band intensities were quantified by ImageJ software (https://imagej.nih.gov/ij), and the data were analyzed in Prism 5.0 software (GraphPad) using a Hill equation binding model.

### Histone deposition assays

TSPYL5-NLD WT and HBM (1 + 2) mutant along with histones H3/H4 were incubated with 146 bp Widom 601 sequence DNA (64) in tetrasome assembly buffer (10 mM Hepes [pH 7.5], 200 mM NaCl, 1 mM TCEP, 5% glycerol, and 0.5 mM EDTA). The samples were incubated at 25 °C for 30 min before analysis on a 6% native gel using 1× TAE (buffer solution with a mixture of Tris base, acetic acid, and EDTA) as running buffer and stained in GelGreen.

### Plasmid supercoiling assay

Histone H3/H4 were incubated with increasing concentrations of TSPYL5-NLD in supercoiling assay buffer (10 mM Hepes [pH 7.5], 150 mM NaCl, 0.5 mM EDTA, 10% glycerol, and 10 mg/ml BSA) at 37 °C for 30 min. pBlueScript supercoiled plasmid DNA (65) was also treated with topoisomerase I (Thermo Fisher Scientific; catalog no.: 38042024) for 30 min separately. Topoisomerase-treated relaxed plasmid DNA was added to the TSPYL5-NLD–H3/H4 mix and incubated for an additional 1 h at 25 °C. Reactions were quenched by adding an equal volume of stop buffer (25% glycerol, 60 mM Tris–HCl, pH 8.0, 30 mM EDTA, and 2% SDS) and incubated for another 30 min. Samples were resolved by electrophoresis in 1.0% agarose gel followed by GelGreen staining.

### Protein structural modeling

Structural modeling of the TSPYL5-NLD was done by the AlphaFold package, which utilized the machine-based deep learning algorithm to design the model (66, 67). Similar model building software RoseTTAFold (68) was also used for structural modeling of the TSPYL5-NLD. The crystal structure of SET/TAF-1B (PDB ID: 2E50) obtained from the RCSB PDB was used for superimposition analysis. The structural figures were created using PYMOL (Schrödinger, LLC).

### Phylogenetic tree analysis

Amino acid sequences of human NAP family proteins (NAP1-like, SET/TAF-1β, TSPY, and TSPY-like family proteins) were retrieved from the UniProt database in FASTA format. Multiple sequence alignments using the MUSCLE program in MEGAX (69) software package were performed, and these aligned sequences were used to generate the phylogenetic tree. The tree depicting evolutionary history was generated by the maximum likelihood method and JTT matrix–based model (70) by applying a total of 250 bootstrap iterations. The generated tree was exported, and the figure for the phylogenetic tree was prepared using Fig-Tree software (http://tree.bio.ed.ac.uk/software/figtree/). Structure-based sequence alignment of human TSPYL5 with SET/TAF-1β and NAP1 proteins (from different organisms representing different phyla) were done using Espript3 (71), where the structure of SET/TAF-1β (PDB: 2E50) was used a template.

## Data availability

All relevant data are included in this article.

## Conflict of interest

The authors declare that they have no conflicts of interest with the contents of this article.
